# Challenges in the cultural adaptation of the German Myeloma Patient Outcome Scale (MyPOS): an outcome measure to support routine symptom assessment in myeloma care

**DOI:** 10.1186/s12885-020-06730-7

**Published:** 2020-03-23

**Authors:** Christina Gerlach, Katherine Taylor, Marion Ferner, Markus Munder, Martin Weber, Christina Ramsenthaler

**Affiliations:** 1 Interdisciplinary Palliative Care Unit, III Department of Medicine, University Medical Center of the Johannes Gutenberg University of Mainz, Mainz, Germany; 2 University Center for Tumour Diseases (uct), University Medical Center of the Johannes Gutenberg University of Mainz, Mainz, Germany; 3 Institute of Medical Biometrics, Epidemiology and Informatics, University Medical Center of the Johannes Gutenberg University of Mainz, Mainz, Germany; 4 III Department of Medicine, Hematology, Medical Oncology and Pneumology, University Medical Center of the Johannes Gutenberg University of Mainz, Mainz, Germany; 5 Department of Palliative care, Policy and Rehabilitation, King’s College London, Florence Nightingale School of Nursing and Midwifery, Cicely Saunders Institute, London, UK; 6 Institute for Sport and Sport Science, Faculty of Economics and Behavioral Sciences, Albert-Ludwigs University of Freiburg, Freiburg i. Br, Germany

**Keywords:** Multiple myeloma, Haematological malignancy, Cultural adaptation, Supportive care, Patient-reported outcome measurement, Quality of life

## Abstract

**Background:**

Patients with multiple myeloma report more problems with quality of life (QoL) than other haematological malignancies over the course of their incurable illness. The patient-centred Myeloma Patient Outcome Scale (MyPOS) was developed to assess and monitor symptoms and supportive care factors in routine care. Our aim was to translate and culturally adapt the outcome measure to the German context, and to explore its face and content validity.

**Methods:**

Translation and cultural adaptation following established guidelines used an exploratory, sequential mixed method study design. Steps included: (1) forward translation to German; (2) backward translation to English; (3) expert review; (4) focus groups with the target population (patients, family members, healthcare professionals) to achieve conceptual equivalence; (5) cognitive interviews using Tourangeau’s model with think-aloud technique to evaluate comprehension and acceptability; (6) final review. Results were analysed using thematic analysis.

**Results:**

Cultural and linguistic differences were noted between the German and English original version. The focus groups (*n* = 11) and cognitive interviews (*n* = 9) both highlighted the need for adapting individual items and their answer options to the German healthcare context. Greater individuality regarding need for information with the right to not be informed was elaborated by patients. While the comprehensive nature of the tool was appreciated, item wording regarding satisfaction with healthcare was deemed not appropriate in the German context. Before implementation into routine care, patients’ concerns about keeping their MyPOS data confidential need to be addressed as a barrier, whereas the MyPOS itself was perceived as a facilitator/prompt for a patient-centred discussion of QoL issues.

**Conclusion:**

With adaptations to answer options and certain items, the German version of the MyPOS can help monitor symptoms and problems afflicting myeloma patients over the course of the disease trajectory. It can help promote a model of comprehensive supportive and patient-centred care for these patients.

## Background

With the ageing of society and an increasing incidence [1–3], cancer is a major public health concern. Haematological cancers and multiple myeloma (MM) in particular exemplify this changing face of cancer with conditions whose management resembles that of a chronic illness, with recurrent treatment patterns followed by maintenance therapy [4, 5]. MM is an incurable cancer of the bone marrow characterised by bone destruction, bone marrow failure with anaemia, immune deficiency, and renal insufficiency [6]. It belongs to the heterogeneous group of plasma cell dyscrasias, which vary from asymptomatic forms to malignant disease with severe end-organ damage and high patient morbidity [7]. Front-line treatment with high-dose chemotherapy and hematopoietic stem cell transplant (HSCT) has improved the median survival for those under the age of 65 to 5 years or longer after a myeloma diagnosis [8]. However, due to its incurability, patients must cope with the incrementally progressive nature of the disease, interspersed with intervals of stable disease and maintenance treatment, while also experiencing long-lasting effects of previous treatments [9, 10]. Moreover, the median age at diagnosis is 69 years, with more than 60% of multiple myeloma patients aged 65 years or older at diagnosis. Due to the aging baby boomer generation, an increase in the proportion of the general population older than 65 years of age is expected between 2020 and 2030. Therefore, in tandem with new and better treatment options, the incidence and prevalence, and subsequently also deaths due to multiple myeloma, will inevitably increase [3].

Therefore, MM patients represent a group of older, haematological cancer patients faced with complex treatment pathways, a long duration of a chronic, yet life-threatening disease with repeated relapses and high levels of uncertainty around disease and treatment progression [11–13]. This results in a high prevalence of physical and psychosocial symptoms and problems throughout the disease trajectory. MM patients may suffer more symptoms and problems than other patients with haematological malignancies. On average, several cross-sectional surveys have shown a mean of 5.6 symptoms, of which 2.3 were rated as severe [9]. A recent meta-analysis showed high prevalence rates for pain and fatigue as well as limitations in aspects of quality of life (QoL) such as physical, role, and social functions [14].

Haematological and psychosocial care of MM patients could be improved by incorporating a longitudinal assessment of symptoms and QoL into routine clinical practice. The routine use of QoL measures allows monitoring of symptoms/QoL, thus leading to better symptom control, improved communication, and higher patient satisfaction [15, 16]. However, few measures have been designed for monitoring QoL in the routine clinical setting; a systematic review of 13 generic and disease-specific health-related QoL measures for multiple myeloma found no single tool developed or validated specifically for routine clinical care [17]. The most common measures, the Functional Assessment of Cancer Therapy-Multiple Myeloma questionnaire (FACT-MM) [18], the EORTC QLQ-MY20 [19], and the M. D. Anderson Cancer Centre Multiple Myeloma measure (MDASI-MM) [20] are disease-specific measures that have been developed for use in clinical trials. Currently, only the EORTC QLQ-MY20 is available in German [21].

The Myeloma Patient Outcome Scale (MyPOS) is a questionnaire developed and validated specifically to measure disease-specific QoL in patients with MM in a routine clinical setting and for monitoring purposes. The initial validation comprised 380 patients at different disease stages in the UK [22]. The MyPOS is a module of the Integrated Palliative/Patient care Outcome Scale (IPOS) [23–25], a short, multidimensional questionnaire to assess palliative care concerns in patients with advanced disease. The MyPOS takes the core items of the POS and extends them with myeloma-specific concerns. The MyPOS comprises a list of 13 symptoms and 20 QoL items which are scored on a 5-point Likert scale and summed into a total score and three subscale scores. Content and construct validity as well as reliability of the MyPOS have been established in clinically representative samples of all disease stages [22, 26, 27]. Its reliability and responsiveness in longitudinal monitoring of QoL have been shown to be satisfactory to good [27].

However, the conceptualization of QoL for patients with MM used in the original MyPOS is UK-focused and must be checked before a German translation of the instrument can be used in practice. Differences in care processes, treatment pathways and cultural background may affect its cross-cultural equivalence [28, 29]. We therefore aimed to explore the cultural, linguistic, and contextual issues during the adaptation of the MyPOS to the German context. Our aim was to translate and cross-culturally adapt the MyPOS and establish its content and face validity for monitoring QoL in MM patients.

## Methods

**Fig. 1:**
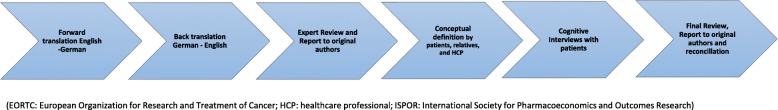
Six-step process of translation and cross-cultural adaptation of the MyPOS based on EORTC and ISPOR standards [33, 34]. (EORTC: European Organization for Research and Treatment of Cancer; HCP: healthcare professional; ISPOR: International Society for Pharmacoeconomics and Outcomes Research)

This study used a multi-step, explorative and sequential mixed-methods design [30, 31]. The procedure for the translation and cultural adaptation of the MyPOS followed the guidelines reported in the manual for cross-cultural adaptation and validation of the parent’s measure POS development group [32], based on commonly accepted standards for cross-cultural adaptation and psychometric testing by the European Organization for Research and Treatment of Cancer (EORTC) and the International Society for Pharmacoeconomics and Outcomes Research (ISPOR) [33, 34]. Because there are already German translation versions of the POS and the IPOS and therefore challenges with adapting items to the German language and healthcare context are already known, we tailored the proposed, six-step process towards supporting a wider exploration of issues of cultural equivalence instead of focusing on psychometric evaluation (see Fig. 1). The cultural adaptation of MyPOS was further informed by the Cultural Equivalency Model for Translating and Adapting Instruments [35], focusing on conceptual (Does the content relate to constructs in the culture?), content (Is the content of each item relevant?), semantic (Is the meaning of the item’s wording the same?), and technical equivalence (Do layout, format, and answer options work the same way?).

### Initial phases: translation, conceptual equivalence, focus groups, and expert review

***Conceptual definitions*** and equivalence of key concepts can be identified by an array of methods. Given the nature of haematological care within Germany, we opted to hold focus groups with the target population, consisting of patients, family members, and healthcare professionals (HCP) to discuss cultural equivalence of key concepts. The combined use of cognitive interviews and focus groups is the recommended best practice for establishing content validity in both new and existing patient-reported outcome measures [36]. The combined use of these methods helps confirm the validity of results through triangulation [37].

Two focus groups, one with patients and their family members (*n* = 5) and one with HCPs (*n* = 6), were held. Recruitment for the patient and family member focus group followed same eligibility criteria and procedures as specified below for cognitive interviews (phase 5). All groups were chaired by CG and were audio recorded. The topic guide was partly based on the topic guides used in the development of IPOS [25] and MyPOS [38] and included discussions about what constitutes QoL in multiple myeloma, the dimensions of QoL, feedback on MyPOS as a measure, aspects of layout, and feedback on MyPOS single items (see Additional file 2). The clinical value of QoL questionnaires was also discussed as well as possible ways in which these measures could be implemented into routine myeloma care [39].

The discussion of cultural equivalence was preceded by the translation of MyPOS to provide a base for feedback on MyPOS. ***Forward translation*** (phase 1) of the MyPOS was performed by two independent, native German speakers from different backgrounds (a medical student proficient and fluent in English and a palliative care clinician). Both translations were reviewed for discrepancies. Consensus was reached by discussion with a third, independent researcher not involved in the initial forward translation. MyPOS as a myeloma-specific module includes the original IPOS items that have already been validated in German [25, 40]. The translated items were used but checked by both translators. The ***backward translation*** (phase 2) was carried out by two native English speakers who were blinded to the original English version. They independently back-translated the questionnaire from German to English. One back translator had a nursing background and patient experience, and the other translator had an epidemiology background. Again, discrepancies were reviewed and resolved by consensus discussions with a third, independent researcher. A record of all items and aspects challenging conceptual, semantic, content, and cultural equivalence was kept and discussed in an ***expert review*** (phase 3). The multidisciplinary group included researchers with knowledge in haemato-oncology and palliative care, all the translators, and a statistician/psychometrician (CR). Based on the discrepancies noted during the translation process, a consensus on content, instructions, wording of items, and response options was reached. We used criteria specified by Koller et al. [41] to characterise changes made during this process of reconciliation. The consolidated version was then pre-tested in ***cognitive interviews*** (phase 5).

### Phase 5: cognitive interviews

Cognitive interviews (*n* = 9) were performed in a separate sample of myeloma patients. As recommended by guidelines [42, 43], these semi-structured interviews served the purpose of further testing the translated and culturally adapted version by using verbal probes and recording cognitive processes (think-aloud) during completion of the questionnaire. Cognitive interviews were supported by a topic guide (see Additional file 3) and complemented the focus group discussions (phase 4) in terms of content. They were based on Tourangeau’s four-stage question response model [44], as adapted for cognitive interviewing by Willis [30, 42]: comprehension, retrieval, judgement, and response formulation. The interviews addressed the patient’s comprehension of the instructions, items and response options, clarity and layout of MyPOS, its length, and difficulties in understanding and answering the questions. Participants were asked to elaborate on reasons why items were perceived as difficult, and to make suggestions as to how instructions, items, and response options could be rewritten to achieve better clarity. Verbal probes concerned missing aspects of QoL in the MyPOS, its acceptability and clinical utility, and the burden associated with its completion.

Patients were recruited from the haematology department of the university hospital, local private practices, and the regional self-help group (LHRM, Leukämiehilfe Rhein-Main). Patients were approached individually, by their treating physician, and via an information leaflet in outpatient clinics. Inclusion criteria were selected in order to approach patients in need of myeloma treatment: a confirmed diagnosis of multiple myeloma (MM) Salmon & Durie stage III, high-risk smoldering MM with positive CRAB-criteria (hyperCalcemia, Renal failure, Anemia, Bone lesions) [6], and any other MM stage triggering supportive therapy. Patients had to be age 18 years or older, sufficiently fluent in written and spoken German, and had to have the capacity to give written informed consent. Exclusion criteria were those too unwell or distressed to participate as judged by their clinical team; any cognitive or communication impairment; and being close to death within the next couple of days. Participants were purposively sampled to achieve maximum variation across the key characteristics: gender, age (</≥ 65 years), stage of disease, and Australia-modified Karnofsky Performance Status (AKPS) [45] with low (≤ 60%), medium (70 and 80%) and high (≥90%) performance status.

Eligible patients were provided with written information about the study and, if willing to participate, gave written informed consent. Interviews were audio-recorded and transcribed verbatim completely for focus groups; cognitive interview quotes were analysed from the interview records.

### Data analysis

Interviews were analysed using thematic content analysis for the development and testing of survey questionnaires [37, 46]. For the focus groups, transcripts were analysed based on an initial coding frame developed by CG. Themes regarding missing QoL aspects in the MyPOS, acceptability, clinical utility, and burden were developed inductively from the material. The analysis aimed towards saturation with no new themes emerging, evaluated through a saturation grid [47].

Feedback on instructions, individual items, and answer options of the MyPOS were recorded in a standardised spreadsheet in Excel, with participant numbers across the top and questionnaire items/attributes listed down the left-hand column. The deductive analytical approach followed Tourangeau’s question response model (comprehension, retrieval, judgment, response formulation). The results from focus groups and cognitive interviews were aggregated separately for each item. The analysis was performed by one researcher (CG), discussing discrepancies with a second researcher (CR) to reach consensus.

Written summaries of the results were used for a **final expert review** as guidance for making changes to the questionnaire. Proposals for changes were reported to and approved by the POS development team (**phase 6**).

### Ethical issues

All participants provided written informed consent. The study protocol was approved by the Ethics Committee of the Medical Council of the federal state of Rhineland-Palatine (837.109.17(10943) on 24 Mar 2017).

## Results

### Demographics

A total of 20 participants, *n* = 11 in two focus groups and *n* = 9 in cognitive interviews, were recruited into the study from April to October 2017. Of the eligible patient participants approached, three declined to participate because of a scheduled transplantation (*n* = 1), sepsis (*n* = 1), and time constraints (*n* = 1). Two patients wanted to include their closest relative, resulting in two family members taking part in the first focus group, consisting of 3 women, and 2 men. Six HCPs, a nurse from the haematology department, a hospice nurse working in the community, a physician specialized in haemato-oncology, and three psychologists in psycho-oncology, participated in the second focus group. Another physician and the social worker were scheduled to take part but cancelled at short notice because of patient care obligations. In addition, two psychologists volunteered to take part, resulting in a disproportionate distribution of professions in the HCP focus group.

The demographic background of those patients taking part in focus groups and cognitive interviews represented the typical patient population in terms of age, marital status, and education. However, no patient with a migration background could be recruited. The HCP focus group included one participant with a background other than Christian. Sociodemographic data are presented in Table 1.

**Table 1:** Baseline characteristics of participants for all phases

	Focus group: patients & relatives	Focus group: Healthcare professionals	Cognitive interviews: patients
	(*n* = 5)	(*n* = 6)	(*n* = 9)
**Sex** (Women: men)	3: 2	3: 3	4: 5
**Age**			Median: 63 Range: 55–79
30–39	1		
40–49	0	2	
50–59	2	3	
60–69	1	1	
70–79	1		
**Religious affiliation**	5 Christian	2 Christian; 1 Muslim; 2 none; 1 n.d.	7 Christian; 2 n.d.
**Marital status**			
Married	4	4	6
Widowed	–	–	1
Divorced	–	–	1
Missing	1	2	2
**Education**			
Secondary school graduate	3	–	2
Technical qualification	–	2	4
University degree	2	2	1
Ph.D. and higher degree	–	2	–
Missing	–	–	2
**Professional experience** (years)		16–33	
**Time since diagnosis (years)**	–	–	Median: 1 Range: 0.5–10
First line treatment	–	–	4
Plateau phase	2 after HSCT	–	3
Relapsed after transplantation	1	–	1
Progressive disease	–	–	1
**Performance status (AKPS)**			
60%	–	–	2
70%	–	–	4
80%	–	–	1
90%	–	–	2

*AKPS* Australia modified Karnofsky Performance Status, *HSCT* Hematopoietic stem cell transplantation, Ph.D.: Philosophical Doctorate, n: sample size; n.d.: no data

The duration of the focus groups was 60–150 min. Time to complete the MyPOS was 10–15 min on average. The mean duration of the cognitive interviews was 54 min (minimum 24 min, maximum 100 min), and took place in a meeting room of the palliative care department (*n* = 3), at a private practice (*n* = 2), on the transplantation ward (*n* = 2), and at the patient’s home (*n* = 2).

The presentation of results follows the cultural equivalency model with the dimensions conceptual, content, semantic, and technical equivalence. The original items with results pooled from focus groups, expert review, and interviews and potential revision of an item or aspect of the questionnaire are shown in Table 2.

**Table 2:** Issues regarding MyPOS items identified in focus groups (*n* = 11) and cognitive interviews (*n* = 9)

#	Original MyPOS item	Subscale/ original POS item?	Issues	Item revised?
1	What have been your main problems and concerns at the moment?	−/ POS	The patients wished for a free text field instead of the original enumerated list, and the question line was shifted into the free-text field in order to avoid patients overlooking the question.	Yes
2	Below is a list of symptoms, which you may or may not have experienced. For each symptom please tick one box that best describes how it has affected you over the past week.		Patients differentiated between problems associated with illness and with therapy with a tendency to overlook therapy side effects. In order to clarify, we added “Below is a list of symptoms and side effects”.	Yes
			The time frame of 1 week was perceived to be too short by patients and HCP. Patients reported about serious issues that happened sometime in the past - severity of the issue was the pivotal factor rather than point of time. Experts discussed accepting that patients may also report issues from the past still relevant to their current situation to inform the consultation.	No
	Pain	Symptoms/ POS	Good comprehension, retrieval, judgement, and response choice by all patients.	No
	Shortness of breath	Symptoms/ POS	Good comprehension, retrieval, judgement, and response choice by all patients.	No
	Weakness or lack of energy	Symptoms/ POS	Good comprehension, retrieval, judgement, and response choice by all patients.	No
	Nausea (feeling like you are going to be sick)	Symptoms/ POS	Good comprehension, retrieval, judgement, and response choice by all patients.	No
	Vomiting (being sick)	Symptoms/ POS	Good comprehension, retrieval, judgement, and response choice by all patients.	No
	Appetite loss	Symptoms/ POS	Good comprehension, retrieval, judgement, and response choice by all patients.	No
	Constipation	Symptoms/ POS	Good comprehension, retrieval, judgement, and response choice by all patients.	No
	Sore or dry mouth	Symptoms/ POS	Good comprehension, retrieval, judgement, and response choice by all patients.	No
	Drowsiness	Symptoms/ POS	One patient wanted to add sleeping problems, which she discovered to do in the list thereafter.	No
	Poor mobility	Symptoms/ POS	Good comprehension, retrieval, judgement, and response choice by all patients.	No
	Diarrhoea	Symptoms	Good comprehension, retrieval, judgement, and response choice by all patients.	No
	Tingling in hands/feet	Symptoms	Good comprehension, retrieval, judgement, and response choice by all patients.	No
	Problems remembering	Symptoms	One patient differentiated between problems remembering things and concentration problems. This was not addressed by other focus group members.	No.
	Over the past week…			
3	Have you been feeling anxious or worried about your illness and treatment?	Emotions/ POS	Expert discussion whether to delete “illness” or both “illness and treatment” because of content overload and Q17 also addressing illness worries. Some patients found that worries about treatment differed from illness. The parent instrument developer team confirmed deletion of “illness” from the German Q3.	Yes
4	Have any of your family or friends been anxious or worried about you?	Emotions/ POS	Good comprehension, retrieval, judgement, and response choice by all patients.	No
5	Have you been feeling depressed?	Emotions/ POS	Good comprehension, retrieval, judgement, and response choice by all patients.	No
6	Have you felt at peace?	Emotions/ POS	Good comprehension, retrieval, judgement, and response choice by all patients.	No
7	Have you been able to share how you are feeling with your family or friends as much as you wanted?	Emotions/ POS	Good comprehension, retrieval, judgement, and response choice by all patients.	No
8	Have you had as much information as you wanted?	Emotions/ POS	Patients from the German population and the generation/ age of typical myeloma manifestation may prefer to receive no or less information in general or currently. Further testing involved showing patients the former POS answer options for that item. All but one patient preferred the latter.	Yes
9	Have any practical problems resulting from your illness been addressed? (such as financial or personal)	Emotions/ POS	This item proved to be the most challenging in the translation. Although most patients understood it well and reported no problems during cognitive interviews, there was a high potential for misunderstanding with this item. Participants proposed asking this question in a simple and more direct way. The consented adaptation now reads: ‘Do you wish you had support for practical problems that have arisen from your disease (e.g. financial or personal issues)?’	Yes
10	Have you been able to carry out your usual activities without help from others?	Emotions	Good comprehension, retrieval, judgement, and response choice by all patients.	No
11	Have you been able to pursue your hobbies and leisure activities?	Emotions	Good comprehension, retrieval, judgement, and response choice by all patients.	No
12	Have you been able to spend quality time with family and friends?	Emotions	Good comprehension, retrieval, judgement, and response choice by all patients.	No
	We would like you to answer this question whether or not you are sexually active. If you prefer not to answer then please tick here:	Emotions	Good comprehension, retrieval, judgement, and response choice by all patients.	No
13	Have you been worrying about your sex life?	Emotions	Good comprehension, retrieval, judgement, and response choice by all patients.	No
14	Have you been worrying about infections?	Emotions	Good comprehension, retrieval, judgement, and response choice by all patients.	No
15	Have you been worrying about your physical appearance?	Emotions	Good comprehension, retrieval, judgement, and response choice by all patients.	No
16	Have you been worrying about your financial situation?	Emotions	Good comprehension, retrieval, judgement, and response choice by all patients.	No
17	Have you been worrying that your illness will get worse?	Emotions	Good comprehension, retrieval, judgement, and response choice by all patients.	No
18	Have you felt able to cope with your illness and treatment?	Emotions	Good comprehension, retrieval, judgement, and response choice by all patients.	No
19	Are you able to contact your doctors or nurses for advice if needed?	Support	Interviewed patients, family, and staff argued for further differentiating these three questions. It was felt that their content does not directly address the patients’ QoL. Patients found doctors and nurses should be asked about separately. However, they were concerned about inadvertently blaming members of the health care profession: patients meet a high number of HCP and the questions ask to make a judgment for this whole group, contrary to the real-world situation in which patients might only have had one bad encounter. Overall, these concerns could very well lead to diminished acceptance of the MyPOS with patients and their families as well as HCP and lead to non-responses. Therefore, these items were removed.	Yes
20	Do your doctors and nurses show a good standard of knowledge and skill when treating you?	Support		Yes
21	Do your doctors and nurses show care and respect when treating you?	Support		Yes
22	Do you have enough information about what might happen to you in the future?	Emotions	Good comprehension, retrieval, judgement, and response choice by all patients.	No
End of question-naire			Some patients wished for a free-text field at the end of the questionnaire due to this being the place to expect them. A note referring to the free-text option at the beginning of MyPOS would help preserve the open-ended focus on the patient’s main problems at the beginning of MyPOS and facilitates the use of the free-text field for issues important to the patient but not addressed in the questionnaire.	Yes

### Conceptual equivalence

Overall, patients in cognitive interviews and focus groups, their family members, and HCPs all confirmed that the MyPOS is a comprehensive measure and includes all items relevant to capture disease-specific QoL in multiple myeloma. No patient or family member felt that important aspects of the QoL experience were missing from the questionnaire. Regarding redundant or unnecessary items, items Q19 to Q21, *advice, knowledge, care and respect from doctors and nurses*, were identified by patients and HCPs as not belonging to the QoL construct, but rather measuring patient satisfaction. This was interpreted to be a separate issue. These questions were also felt to hamper return of the MyPOS due to these questions asking the respondent to make a global judgment of the whole HCP group. Patients feared that such feedback could be perceived as a criticism of their HCPs and could make them seem ungrateful for their care. This led to concerns regarding the confidentiality of the questionnaire and its use in daily clinical practice. It was also advised to enable separate judgments regarding doctors and nurses. After pooling results from all phases, it was decided to remove Q19 to Q21 from the German translation in concordance with the POS development team.

### Content and semantic equivalence

In the expert review and cognitive interviews, the instructions for Question 2 were criticized. These instructions specify that the following list of symptoms should be scored according to how much the patient had been affected by each symptom in the past week. Patients were found to be at risk for underreporting subsequent symptoms as they distinguished between symptoms arising from their illness and symptoms arising from their treatment. It was decided that the instructions needed to be more precise, and the phrase “symptoms and side effects” was added to the instructions.

Questions 8 and 22, *having as much information as wanted* and *having enough information about what might happen in the future*, posed problems in the translation process because of a lack of answer options for patients preferring less information. HCPs in the focus group further elaborated on patient-centred care also entailing honouring patients’ and families’ wishes for less information: *“No, but it’s about killing people with information, and I think that’s a legitimate question, because people hear what kind of disease they have, and then they’re fed tens of thousands of pieces of information that go right in and out anyway, right?*” (HCP 2, nurse). Following the discussion around answer options and information needs, patients in the focus groups also suggested adding to the instructions for completion of the MyPOS and making it clearer that patients were allowed to not answer questions if they felt concerned or unable to deal with a problem. Although the MyPOS questionnaire ends with instructions for the patient to speak to a member of a clinical team if concerns about any of the issues raised persist, it was felt that these instructions had to be presented earlier in the questionnaire. Patients also proposed to open up the item *information needs* so that it also includes their informal caregivers: ‘Have your family or confidential persons had as much information as they wanted?’

Question 5, *feeling depressed,* challenged semantic equivalence in the sense that the term depression was perceived by patients in the focus groups and cognitive interviews as referring to psychiatric disease. This item had already been translated into German during the cultural adaptation of the parent measure (IPOS). However, the existing translation was deemed inappropriate and containing ambiguous wording. An adapted translation was tested in the cognitive interviews and the item now translates as: ‘*Have you been feeling sad or depressed/gloomy?* ’.

Questions (Q) addressing practical issues and financial worries (Q9 and Q16) were found to be challenging in terms of understanding and were perceived to be intrusive. One patient in particular highlighted this issue during the cognitive interview. A more direct and non-disturbing rephrasing was discussed in the final expert review: ‘Do you wish you had support for practical problems that have arisen from your disease (e.g. financial or personal issues)? ’.

Among the QoL items of the MyPOS, item Q12, *being able to spend quality time with family and friends*, could not be translated verbatim into German as no such concept as quality time exists in the German language. The verbatim translation has a connotation of duration rather than quality of the time spent together. A translation of “spending time together” was perceived as not mirroring the aspect of enjoyment. The proposed revised translation which was cognitively tested therefore specified whether the patient was able to cherish those moments s/he spent with family and friends.

The MyPOS contains an item on sexual well-being (Q13). Patients in the focus groups and cognitive interviews welcomed this question and did not feel uncomfortable or embarrassed answering this. In contrast sexuality was controversial when discussed in the HCP focus group. While the importance of this aspect was not denied, it was also perceived to be an embarrassing issue that could hamper completion of the questionnaire.

“I don’t think patients dare to address it with the doctors. Maybe they also have feelings of shame, which is easier with the nurses. So, you are somehow without fear or favour. It’s a bit more distant with the doctors, I think, there are a lot of questions coming up towards us. Can I have sex with my partner at all? What do I have to pay attention to? Can I hurt her or him? And I think these are very, very big points. Something that also moves the patients, because there are also many younger ones. They’re even afraid to cuddle up with each other, because it’s always said, watch out because of the risk of infection and I think that’s really bad.” (HCP 2, nurse).

### Technical equivalence

The layout of the original questionnaire only needed to be improved for Q1, *main problems and concerns*. Patients advised using a free-text field instead of three separate lines. A box was added to help patients not overlook this item. Response options were perceived to work well, except for items Q8 and Q22, *information needs*, and item Q9, *practical matters*. Following the revision of these items, the answer options were adapted accordingly (see Table 2). Q19 to Q21 in particular generated a discussion on gender issues. German is a language with gender-specific nouns, thus having female and male versions of the English ‘doctor’ and ‘nurse’. Appropriating doctor with ‘he’ and nurse with ‘she’ conveys an outdated gender stereotype. However, mentioning both the female and male version can make the text cluttered. It was therefore opted to include the general term for healthcare staff.

The original time frame of the MyPOS is asking about the past week. Different alternative time frames were discussed in the patient focus group and the cognitive interviews. It was noted that while a time frame of 1 week might work well with physical symptoms, emotional issues might need a longer time frame. Patients also reported that severe symptoms that happened to be beyond the time frame of 1 week would be reported nonetheless. After much discussion, the one-week time frame was concluded to be the most acceptable.

Due to polyneuropathy, patients preferred a paper/pencil version of the MyPOS presented on a clipboard rather than a tablet computer version of the questionnaire. They felt that completion before a clinical visit would help them identify the critical issues they would like to discuss with their doctor. The tool was perceived as an aid but not as a substitute for the consultation. Patients in both focus groups and cognitive interviews advocated for developing a caregiver-reported version of the MyPOS. The MyPOS is presented in Additional file 1. The original English and the revised German version of the MyPOS are available for download at https://pos-pal.org/.

## Discussion

In this study, we translated and culturally adapted the Myeloma Patient Outcome Scale, a patient-reported symptom and QoL tool to support monitoring of patient-centred issues in routine clinical practice. The MyPOS is the first disease-specific myeloma questionnaire available in German that supports both clinical assessment/monitoring as well as use within research studies. The only other available translation of a health-related QoL questionnaire, the EORTC QLQ-MY20 only offers utility within research [21]. The German translation of the MyPOS has been shown to possess content/face validity and acceptability for patients and staff. However, certain adaptations honouring patient-centred wishes for more or less information, separating patient satisfaction from QoL, and involving family members in the assessment process were needed to achieve cultural equivalence within a German healthcare context. The questionnaire was perceived as clinically important in preparing and guiding individual clinical encounters with HCPs and helping patients raise embarrassing or challenging issues with their HCPs. MyPOS may help to direct and focus conversations when employed longitudinally. The sequential use of focus groups to establish conceptual equivalence and cognitive interviews to cognitively test individual items benefitted the content validity by stressing separate issues regarding the comprehension and utility of the questionnaire.

Several of the issues regarding comprehension and equivalence of individual items observed in this study have also been reported in recent cultural adaptation studies of the parent measure, the IPOS. Our study also found more problems with the adaptation of items from the parent measure than from the myeloma-part of the measure. Comprehension issues consistently reported in the literature concern item Q5, *feeling depressed*. The association of the term ‘depressed’ with a medical diagnosis was also noted in a study testing a Swedish version of the IPOS [48]. Similar to our finding, the Swedish research group opted for a colloquial term. They found that this translation was better fitted towards eliciting the patient’s mood. Some authors argue for directly asking patients whether they *think they are depressed* rather than opting for a wording of *feeling depressed*. However, results are inconsistent [49, 50]. Similar findings have been reported in cognitive interviews during the German translation of the IPOS [25] and its Italian translation [51]. The issue around culturally adapting Q9 as well as Q19, both referring to problems of a financial or personal nature being addressed, was also reported in the French and Italian translation of Q9 in the IPOS [51, 52]. Additional problems concerning the response options have been described.

Items in the healthcare support subscale caused the most concerns from a content and face validity point of view. Patients identified issues regarding these questions measuring a construct other than QoL and issues regarding wording, adaptation and the global nature of these questions. Both the cross-sectional and longitudinal validity studies of the English MyPOS [22, 26] reported poor psychometric criteria for these items, with pronounced ceiling effects in the scale and poor reliability. Similar issues were found for a set of healthcare satisfaction items that were part of the original 24-item version of the EORTC QLQ-MY20 [53]. Due to the same reasons, these items were removed from subsequent versions of the questionnaire. Despite patient satisfaction being assigned a central role in patient outcomes [54], it is regarded as a patient experience measure rather than an outcome measure and thus perceived as distinct from QoL as a construct [55]. However, healthcare satisfaction and QoL do seem to overlap. The addition of these items to the original MyPOS was based on findings from the qualitative interviews to develop the measure [38]. In stem cell transplant populations, it has also been reported that patients with higher levels of satisfaction with medical care reported higher levels of QoL, despite ongoing physical and psychosocial morbidity after transplant [56]. The main critique in this German sample centred on worries of negative satisfaction statements sending the wrong message to HCPs and jeopardizing the long-lasting relationships with doctors and nurses who care for these patients throughout their illness trajectory. Therefore, the negative connotation of these items might well point towards different care models and patient experiences with care in the German context.

This less fragmented care model that was reported by patients in our study also serves the clinical utility of the MyPOS. Unlike the MDASI-MM and the EORTC-QLQ-MY20, the MyPOS contains more items regarding worry about the future, information needs, and coping processes as well as adaptation processes, all relevant to and reflecting the prolonged disease trajectory. These issues have also been highlighted as important in recent qualitative studies focusing on the advanced myeloma population [11–13, 57, 58]. The MyPOS was developed as a tool to go beyond the simple assessment of symptom status and asking for an evaluation of physical, emotional, spiritual, and QoL morbidity. Outcome measures to be used in routine clinical care have been shown to be powerful instruments to improve wellbeing and outcomes of cancer patients as well as improve communication with HCPs [15]. These tools cannot replace the interventions required to meet patients’ and their family caregiver’s needs [59, 60], but they can help to identify and monitor high levels of unmet supportive care needs and help integrate those services into care [61, 62]. The interface of supportive care and PC with haematology differs from the one with oncology because MM patients usually have a long and close relationship with the haematology team. LeBlanc & El-Jawahri even proposed unifying PC with haematology [63] by having haemato-oncological staff with a qualification in PC follow a primary PC approach supported by close collaboration with PC specialists when required, as indicated e.g. by patient-reported outcomes and joint case reviews. Porta-Sales et al. demonstrated the benefit of a cooperative PC-haematology approach for outpatients guided by impaired QoL in a retrospective analysis of their Multiple Myeloma Palliative Care Clinic [64]. The MyPOS could support such a care model.

### Methodological limitations

The sample size for this study can be considered a limitation. Both the focus group study as well as the cognitive interviews would have benefitted from a larger number of patients taking part and being interviewed. However, we followed a purposive sampling approach using a sampling matrix to ensure maximum variation. We also aimed to represent the diversity of care providers in the HCP sample, which was challenged due to the high workloads, resulting in a disproportionate representation of psychologists in the HCP sample. Although we managed to sample a group of patients from different age groups, myeloma patients unfit to receive active therapy or at the end of life represented by lower AKPS levels are under-represented in this study. It would also be worthwhile to sample extreme cases to explore aberrant views on the utility of outcome measures in clinical practice. However, we included multiple settings in a hospital, private practice, outpatients and inpatients, including those undergoing haematopoietic stem cell transplantation. HSCT in particular is the first haematology setting where PC was integrated successfully into the care of patients challenged by an uncertain prognosis and high, multidimensional symptom burden [65, 66].

## Conclusion

In this study, we translated and culturally adapted the Myeloma Patient Outcome Scale, a QoL questionnaire to support the assessment and monitoring of symptoms and wellbeing in routine clinical care. After adaptations to the German care context, the MyPOS demonstrated good face and content validity. MyPOS German is now ready to support successful clinical approaches to better meet myeloma patients’ needs for palliation of symptoms as well as psychosocial concerns which impact on QoL. Next steps in its German validation include formal psychometric and clinical utility testing. Further, the demand for a proxy version should be appreciated and addressed, including assessment of informal caregiver burden.

## Supplementary information

**Additional file 1:** Myeloma Patient Outcome Scale (MyPOS) English Version.

**Additional file 2:** Topic guide for focus groups with patients, family, and healthcare professionals about conceptual equivalence, and feedback on MyPOS as a measure, aspects of layout, and feedback on MyPOS single items.

**Additional file 3:** Topic guide for cognitive interviews with patients addressing the patient’s comprehension and layout of MyPOS, suggestions for improvement, its acceptability and clinical utility, and the burden associated with its completion.

## Data Availability

The datasets generated and/or analysed during the current study are not publicly available due to the large size of the transcripts from each interview and sensitivity and specificity of some of the information from the interviews, but the demographic information and some of the de-identified descriptions can be made available upon reasonable request to the corresponding author. The MyPOS questionnaire is available for download at https://pos-pal.org/.

## Abbreviations

AKPS: Australia-modified Karnofsky Performance Status
CRAB-criteria: Hypercalcemia, Renal failure, Anemia, Bone lesions
EORTC QLQ-MY20: European Organization for Research and Treatment of Cancer Quality of Life Questionnaire Myeloma 20
FACT-MM: Functional Assessment of Cancer Therapy-Multiple Myeloma questionnaire
HCP: Healthcare professional
HSCT: Haematopoietic stem cell transplantation
IPOS: Integrated Palliative/Patient care Outcome Scale
ISPOR: International Society for Pharmacoeconomics and Outcomes Research
MDASI-MM: M. D. Anderson Cancer Centre Multiple Myeloma measure
MM: Multiple Myeloma
MyPOS: Myeloma Patient Outcome Scale
PC: Palliative care
UK: United Kingdom
