# Supplementary material for: Challenges in the cultural adaptation of the German Myeloma Patient Outcome Scale (MyPOS): an outcome measure to support routine symptom assessment in myeloma care
Source: BMC Cancer. 2020 Mar 23;20:245. doi: 10.1186/s12885-020-06730-7 (PMC7092563; doi:10.1186/s12885-020-06730-7)
Supplement: Supplementary file 1 — Additional file 1: Myeloma Patient Outcome Scale (MyPOS) English Version. [file 12885_2020_6730_MOESM1_ESM.pdf]

MyPOS Patient Version

www.pos-pal.org

Patient name: ……………………………………………….

Date (dd/mm/yyyy): ……………

Patient number: ………………………………… *(for staff use)*

Please answer the following questions by ticking the box that is most true for you. It is important to answer all of the questions if possible. Your answers will be used to help improve your care and the care of others. Thank you.

Q1. What are your main problems or concerns at the moment?

1.

2.

3.

Q2. Below is a list of symptoms, which you may or may not have experienced. For each symptom please tick one box that best describes how it has affected you over the past week.

	No, not at all	***Slightly***	***Moderately***	***Severely***	***Overwhelmingly***
**Pain**	0 ❑	1 ❑	2 ❑	3 ❑	4 ❑
**Shortness of breath**	0 ❑	1 ❑	2 ❑	3 ❑	4 ❑
**Weakness or lack of energy**	0 ❑	1 ❑	2 ❑	3 ❑	4 ❑
**Nausea (feeling like you are going to be sick)**	0 ❑	1 ❑	2 ❑	3 ❑	4 ❑
**Vomiting (being sick)**	0 ❑	1 ❑	2 ❑	3 ❑	4 ❑
**Poor appetite**	0 ❑	1 ❑	2 ❑	3 ❑	4 ❑
**Constipation**	0 ❑	1 ❑	2 ❑	3 ❑	4 ❑
**Sore or dry mouth**	0 ❑	1 ❑	2 ❑	3 ❑	4 ❑
**Drowsiness**	0 ❑	1 ❑	2 ❑	3 ❑	4 ❑
**Poor mobility**	0 ❑	1 ❑	2 ❑	3 ❑	4 ❑
**Diarrhoea**	0 ❑	1 ❑	2 ❑	3 ❑	4 ❑
**Tingling in the hands and / or feet**	0 ❑	1 ❑	2 ❑	3 ❑	4 ❑
**Difficulty remembering things**	0 ❑	1 ❑	2 ❑	3 ❑	4 ❑

Please list any other symptoms not mentioned above, and tick one box to show how they have affected you over the past week.

**1.**	0 ❑	1 ❑	2 ❑	3 ❑	4 ❑
**2.**	0 ❑	1 ❑	2 ❑	3 ❑	4 ❑
**3.**	0 ❑	1 ❑	2 ❑	3 ❑	4 ❑

		No, not at all	Occasionally	Sometimes	Most of the time	Yes, always
**Q3.**	**Over the past week, have you been feeling anxious or worried about your illness or treatment?**	0 ❑	1 ❑	2 ❑	3 ❑	4 ❑
**Q4.**	**Over the past week, have any of your family or friends been anxious or worried about you?**	0 ❑	1 ❑	2 ❑	3 ❑	4 ❑
**Q5.**	**Over the past week, have you been feeling depressed?**	0 ❑	1 ❑	2 ❑	3 ❑	4 ❑

		Yes, always	Most of the time	Sometimes	Occasionally	No, not at all
**Q6.**	**Over the past week, have you felt at peace?**	0 ❑	1 ❑	2 ❑	3 ❑	4 ❑

		Yes, as much as I wanted	Most of the time	Sometimes	Occasionally	No, not at all
**Q7.**	**Over the past week, have you been able to share how you are feeling with your family or friends?**	0 ❑	1 ❑	2 ❑	3 ❑	4 ❑

		Enough information<br>the right amount for me	Information received<br>but hard to understand	Information received<br>but would like more	Very little information<br>and would like more	No information received<br>and would like information
**Q8.**	**Over the past week, have you had as much information as you wanted?**	0 ❑	1 ❑	2 ❑	3 ❑	4 ❑

		No problems/ Problems addressed	Problems being addressed	Problems partly addressed	Most problems not addressed	Problems not addressed at all
**Q9.**	**Over the past week, have any practical matters resulting from your illness been addressed? (such as financial or personal)**	0 ❑	1 ❑	2 ❑	3 ❑	4 ❑

Please turn to the next page.

	Yes, as much as I wanted	Most of the time	Sometimes	Occasionally	No, not at all
**Q10. Over the past week, have you been able to carry out your usual activities without help from others?**	0 ❑	1 ❑	2 ❑	3 ❑	4 ❑
**Q11. Over the past week, have you been able to pursue your hobbies and leisure activities?**	0 ❑	1 ❑	2 ❑	3 ❑	4 ❑
**Q12. Over the past week, have you been able to spend quality time with family and friends?**	0 ❑	1 ❑	2 ❑	3 ❑	4 ❑

We would like you to answer this question whether or not you are sexually active

Or if you would prefer not to answer then please tick here: ❑

	No, not at all	Occasionally	Sometimes	Most of the time	Yes, always
**Q13. Over the past week, have you been worrying about your sex life?**	0 ❑	1 ❑	2 ❑	3 ❑	4 ❑

	No, not at all	Occasionally	Sometimes	Most of the time	Yes, always
**Q14. Over the past week, have you been worrying about infections?**	0 ❑	1 ❑	2 ❑	3 ❑	4 ❑
**Q15. Over the past week, have you been worrying about your physical appearance?**	0 ❑	1 ❑	2 ❑	3 ❑	4 ❑
**Q16. Over the past week, have you been worrying about your financial situation?**	0 ❑	1 ❑	2 ❑	3 ❑	4 ❑
**Q17. Over the past week, have you been worrying that your illness will get worse?**	0 ❑	1 ❑	2 ❑	3 ❑	4 ❑

	Yes, always	Most of the time	Sometimes	Occasionally	No, not at all
**Q18. Over the past week, have you felt able to cope with your illness and treatment?**	0 ❑	1 ❑	2 ❑	3 ❑	4 ❑
**Q19. Are you able to contact your doctors or nurses for advice if needed?**	0 ❑	1 ❑	2 ❑	3 ❑	4 ❑
**Q20. Do your doctors and nurses show a good standard of knowledge and skill when treating you?**	0 ❑	1 ❑	2 ❑	3 ❑	4 ❑
**Q21. Do your doctors and nurses show care and respect when treating you?**	0 ❑	1 ❑	2 ❑	3 ❑	4 ❑

	Enough information the right amount for me	Information received but hard to understand	Information received but would like more	Very little information and would like more	No information received and would like information
**Q22. Do you have enough information about what might happen to you in the future?**	0 ❑	1 ❑	2 ❑	3 ❑	4 ❑

	On my own	With help from a friend or relative	With help from a staff member
**Q23. How did you complete this questionnaire?**	❑	❑	❑

Thank you for your time. If you are worried about any of the issues raised on this questionnaire then please speak to your doctor or nurse.
