# Supplementary material for: Challenges in the cultural adaptation of the German Myeloma Patient Outcome Scale (MyPOS): an outcome measure to support routine symptom assessment in myeloma care
Source: BMC Cancer. 2020 Mar 23;20:245. doi: 10.1186/s12885-020-06730-7 (PMC7092563; doi:10.1186/s12885-020-06730-7)
Supplement: Supplementary file 2 — Additional file 2: Topic guide for focus groups with patients, family, and healthcare professionals about conceptual equivalence, and feedback on MyPOS as a measure, aspects of layout, and feedback on MyPOS single items. [file 12885_2020_6730_MOESM2_ESM.pdf]

Topic Guide

Translation, Cross-Cultural Adaptation and Psychometric Testing- MyPOS German
Phase 4: Conceptual Definition or Equivalence
PPI – target population

Objectives/ Ziele:

- Identification of topics and aspects that are important for the QoL of multiple myeloma patients
  Identifizierung von Themen und Aspekten, die für die QoL von Patientinnen und Patienten mit Multiplem Myelom wichtig sind
- Exploration to what extent these concerns are affected by the disease
  Exploration, ob und in wieweit diese Belange durch die Erkrankung beeinträchtigt werden
- Identification of gaps or missing topics in MyPOS
  Identifizierung von Lücken bzw. Fehlenden Themen im MyPOS GERMAN V04

- *Warm up:*
  - Introduce oneself, purpose of the discussion/ *Vorstellung, Zweck des Gespräches*
  - Introduction of participants, motivation to take part/ *TN-Vorstellung, Motivation teilzunehmen*

- *Introduction/ Einleitung:*
  - Interview procedure, recording so that important information is not lost, confidentiality, duration *Ablauf des Interviews , Aufnahme, damit nichts wichtiges verloren geht, von dem was TN mitzuteilen haben, Vertraulichkeit, Dauer*
  - Reasons for recording/ *Erklären, warum Aufnahme auf Band*
  - Due to confidentiality, the participants are not addressed by name, but if this should happen by mistake, the name will not be transcribed./ *Wg. Vertraulichkeit werden TN nicht mit Namen angesprochen, falls das doch versehentlich passieren sollte, wird dieser nicht transkribiert*
  - Interruption or termination by participants possible at any time./ *Unterbrechung oder Abbruch des Gespräches jederzeit möglich.*
  - The right to decline to discuss a particular question/ *Ablehnung, über bestimmte Fragen zu diskutieren erlaubt*
  - There are no „correct“ or „wrong“ answers./ *Es gibt keine „richtigen“ oder „falschen“ Antworten*

  - --- START RECORDING ---

- *QoL:* (evtl. Flipchart)
  - In general, if you are thinking about your quality of life, which issues are important to you?
    „Ganz allgemein: wenn Sie über Ihre Lebensqualität nachdenken – welche Belange sind Ihnen wichtig?“
  - What gives your life special joy, value or quality?
    „Was verleiht Ihrem Leben besondere Freude, Wert oder Qualität?“
  - What affects this quality of life?/ *„Was beeinträchtigt diese Lebensqualität?“*
  - What does good quality of life mean to you?/ *„Was bedeutet Ihnen eine gute Lebensqualität?“*
  - What could improve your QoL/ what could worsen it?
    „Was könnte Ihre Lebensqualität noch verbessern/ was verschlechtern?“

- *Myeloma and QoL:*
  - In what sense did the disease enrich/affect you life?
    „Auf welche Weise, hat die Erkrankung Ihr Leben bereichert?/ beeinträchtigt?”

--- **Pause** ---

- ***MyPOS:***
  - Please read the questionnaire for yourself and fill it out. You don't have to hand in the sheet.
    "Bitte lesen Sie den MyPOS für sich und füllen ihn aus. Sie tun das für sich. Sie müssen den Bogen nicht abgeben."

- ***Relevance:*** - Which of the questions are (not) relevant for your QoL?
  "Welche Fragen sind (ir)relevant für Ihre Lebensqualität?"

- ***Layout:***

  -> SFF

  Structure/*Struktur:*

  1) Lenght/ *Länge*
  2) Font size and type easy to read?/ *Schriftgröße und -type gut lesbar?*
  3) Horizontal or vertical arrangement of response options?
     Horizontale Anordnung der Antwortoptionen oder vertikal?

  Items/ *Fragen:* 1) Time intervall requested: 3 days or one week o.k.? proposals
  Abgefragtes Zeitintervall 3 Tage, 1 Woche o.k.? Vorschlag
  2) More free-text? Why (not)?/ *Mehr Freitext? Warum (nicht)?*

  Scale format/ *Skalenformat:* Likert o.k.?, Number of answer opitons, proposal
  Anzahl der Antwortoptionen/ Vorschlag

- ***Application of questionnaires in clinical routine***
  Anwendung von Fragebögen im klinischen Alltag:
  - What issues do you discuss with your physicians on a regular base?
    Über welche Themen sprechen Sie normalerweise mit Ihrer Ärztin oder Ihrem Arzt?
  - What themes are actually important to you but are rarely or not at all addressed at the clinical encounter?/ *Welche Themen, die Ihnen eigentlich wichtig sind, werden eher seltener oder gar nicht angesprochen?*
  - Do you think that those themes could be caught by a questionnaire?
    Denken Sie, dass diese Themen durch einen Fragebogen aufgefangen werden könnten?
  - What other issues should be included?
    Welche Belange sollten noch in den Fragebogen aufgenommen werden?
  - In your opinion, what is the practical value of MyPOS?
    Welchen praktischen Wert hat der MyPOS aus Ihrer Sicht?
  - *How should the questionnaire be used?/ Wie sollte der Fragebogen eingesetzt werden?*
    Probe: outpatients/ inpatients? At what point of time? Or phase of illness?
    Ambulant/ stationär? Was ist ein geeigneter Zeitpunkt? oder Krankheitsphase?
  - Who do you want to talk to about this?/ *Mit wem würden Sie darüber sprechen wollen?*
  - What do you think about a tablet computer version?/ *Was halten Sie von einer Tablet Version?*

-- **Pause** --

- ***Studien:***

  What are your expectations towards further studies about the QoL in multiple myeloma?
  „Was erwarten Sie von weiteren Studien zur Lebensqualität bei Multiplem Myelom?"
  What should be considered? What should not be forgotten?
  „Was sollte unbedingt beachtet werden?" „Was darf nicht vergessen werden?"

- ***Abschluss***
  (1) What do you like to add?/ *Was möchten Sie außerdem noch ergänzen?*
  (2) Thank you very much for your participation! You make an important contribution to the development of a meaningful and acceptable instrument that will help us improve care for myeloma patients.
  Herzlichen Dank für Ihre Teilnahme! Sie leisten einen wichtigen Beitrag zu Entwicklung eines sinnvollen und akzeptablen Instrumentes, das uns helfen wird die Betreuung von Myelompatienten zu verbessern.

----------------------**THANK YOU! STOP RECORDING Danke! Band stoppen.**--
