# Supplementary material for: Challenges in the cultural adaptation of the German Myeloma Patient Outcome Scale (MyPOS): an outcome measure to support routine symptom assessment in myeloma care
Source: BMC Cancer. 2020 Mar 23;20:245. doi: 10.1186/s12885-020-06730-7 (PMC7092563; doi:10.1186/s12885-020-06730-7)
Supplement: Supplementary file 3 — Additional file 3: Topic guide for cognitive interviews with patients addressing the patient’s comprehension and layout of MyPOS, suggestions for improvement, its acceptability and clinical utility, and the burden associated with its completion. [file 12885_2020_6730_MOESM3_ESM.pdf]

Topic Guide

Cognitive Interviews with multiple myeloma patients using MyPOS

Objective/ *Zielsetzung*:

- To explore the cognitive processes in the responding person during reading, comprehension and answering to MyPOS
  Exploration der kognitiven Prozesse der befragten Personen während der Lesens, Auffassens und Beantwortens der Fragen des MyPOS

- Introduction/ *Einführung:*

  - Welcome (introduce oneself)/ *Begrüßung, (Vorstellung)*
  - Purpose of the study, confidentiality, the right to interrupt or cancel the interview at any time, the right to decline to answer a particular question
    Studienzweck, Vertraulichkeit, Recht jederzeit zu unterbrechen oder abzubrechen, Recht Beantwortung einer Frage abzulehnen

  - I would like to show you the questionnaire we talked about. Please, read and answer the questions one at a time./ Ich zeige Ihnen gleich den Fragebogen, über den wir gesprochen haben. Bitte lesen und beantworten Sie Frage für Frage, eine nach der anderen.
  - We will pause for a moment at each question and talk about it before moving on to the next question./ Wir werden bei jeder Frage kurz innehalten und über die Frage sprechen bevor wir zur nächsten gehen.
  - It is important that you ‚Think out loud' (SHOW IT). Please, speak out what you are thinking while you read and respond to the question./ Es ist wichtig, dass Sie dabei "laut denken". (*VORMACHEN*) Sprechen Sie aus, was in Ihnen vorgeht, während Sie die Fragen lesen und beantworten.
  - I will also ask some questions specific to the questionnaire.
    Ich werde Ihnen zum Fragebogen auch einige konkrete Fragen stellen.

  - Please accept my apologies if the questions occur repeatedly.
    Und bitte entschuldigen Sie, wenn sich meine Fragen wiederholen.

  - The study is not so much about the answer but to *how you arrive to the answer* to the single items – what you are thinking, what the item means to you, and what you are thinking about when you choose your answer option.
    In dieser Studie geht es weniger darum, was Sie antworten als darum *wie Sie zu der Antwort kommen* – was Sie dabei denken, was die Frage bedeutet, und worüber Sie nachdenken, während Sie sich für eine der Antworten entscheiden.

  - You can say everything. I am interested in all your thoughts and opinions.
    Sie dürfen alles sagen. Ich bin an all Ihren Gedanken und Ansichten interessiert.

--------------------------------START RECORD/ AUFNAHME STARTEN-----------------------

------------------MEASURE TIME WHILE FILLING IN/ ZEIT STOPPEN BEIM AUSFÜLLEN--------------

- *__General/__* *__Allgemein:__*

  - What were you thinking about when you answered the question?
    Was haben Sie gedacht als Sie die Frage beantwortet haben?

  - I noticed you hesitated before giving your answer – what were you thinking about then?/ *Mir ist aufgefallen, dass Sie kurz gezögert haben, bevor sie geantwortet haben – worüber haben Sie da nachgedacht?*

- *__Comprehension:__* ***what does the respondent believe the question to be asking?***
  __Verständnis:__ Was denkt die befragte Person wonach die Frage fragt?

  - What does the question mean to you, in your own words?
    Was bedeutet die Frage, in Ihren eigenen Worten?

  - What does the word XXXXXX mean to you? (if certain words are thought to be problematic)/ *Was bedeutet das Wort XXXXXX (nach Ihrem Verständnis)? (wenn bestimmte Worte problematisch erscheinen)*

  - *How easy or difficult was it to understand this question?*
    Wie leicht oder schwer verständlich war die Frage?

  - (If problem) How would you change this question?
    (Falls ein Problem besteht:) Wie würden Sie die Frage verändern?

- *__Retrieval:__* ***could they recall the information required by the question? Was the time frame suitable?***
  __Abruf__: Kann sich die befragte Person an die erfragte Information erinnern? Ist der angegebene Zeitraum geeignet?

  - How well could you remember your experience when answering this question?
    Wie gut konnten Sie sich während der Beantwortung der Frage an Ihre eigene Erfahrung (mit XXX) erinnern?

  - Was it easy or difficult to think about the past (week) when answering this question?/ *War es leicht oder schwierig an die letzte Woche zu denken als Sie die Frage beantwortet haben?*

  - Would there be a different time period that would be easier to understand?
    Wäre es einfacher mit einem anderen Zeitraum?

- *__Judgement:__* ***is the respondent able to make an evaluation based on the information recalled/*** *__Bewertung:__ Ist die befragte Person in der Lage auf Grund der aus der Erinnerung abgerufenen Information eine Einschätzung zu treffen?*

  - What were you thinking about when you answered this question?
    Woran haben Sie gedacht, als Sie die Frage beantwortet haben?

  - How did you arrive at your answer to that question?
    Wie sind Sie zu Ihrer Antwort auf diese Frage gekommen?

- Was that easy or hard to arrive at your answer? – Why?
  Fanden Sie es leicht oder schwer die Frage zu beantworten? - Wieso?

- <u>*Response:*</u> ***is the respondent able to map their internally generated answer to a response option?/*** <u>*Wiedergabe:*</u> *Ist die befragte Person fähig ihre innerlich generierte Antwort einer der Antwortoptionen zuzuordnen?*

  - How did you choose your answer to this question?
  Wie haben Sie Ihre Antwort auf diese Frage ausgewählt?

  - Was it hard or easy to select an answer from the options given?
  War es schwer oder leicht eine der Antwortoptionen auszusuchen?

  - Did all options make sense for this question?
  Sind alle Antwortoptionen für diese Frage sinnvoll?

  - Can you think of another answer option you can't find here?
  Fällt Ihnen noch eine weitere Antwortoption ein, die Sie hier nicht finden?

---QUESTIONS AFTER FILLING IN/ZIELGERICHTET FRAGEN NACH AUSFÜLLEN DES BOGENS-----

- <u>*Application of questionnaires in the clinical routine*</u>
  <u>*Anwendung von Fragebögen im klinischen Alltag:*</u>

  - Reading and responding to the questions can trigger reactions. How did you feel about it?*/ Das Lesen und Beantworten der Fragen kann in einem verschiedene Reaktionen auslösen. Wie war es bei Ihnen?*
  -
  - What issues do you discuss with your physicians on a regular base?
    Über welche Themen sprechen Sie normalerweise mit Ihrer Ärztin oder Ihrem Arzt?
  - What themes are actually important to you but are rarely or not at all addressed at the clinical encounter?*/ Welche Themen, die Ihnen eigentlich wichtig sind, werden eher seltener oder gar nicht angesprochen?*
  - Do you think that those themes could be caught by a questionnaire?
    Denken Sie, dass diese Themen durch einen Fragebogen aufgefangen werden könnten?

  - What other issues should be included?
    Welche Belange sollten noch in den Fragebogen aufgenommen werden?

  - In your opinion, what is the practical value of MyPOS?
    Welchen praktischen Wert hat der MyPOS aus Ihrer Sicht?

  - How should the questionnaire be used?*/ Wie sollte der Fragebogen eingesetzt werden?*
  - Who do you want to talk to about this?*/ Mit wem würden Sie darüber sprechen wollen?*

  - *What do you think about a tablet computer version?*
    Was halten Sie von einer Tablet Version?
  - What do you think about the lengths of the questionnaire?
    Was denken Sie zu der Länge des Fragebogens?

- ***<u>Other/ Sonstiges:</u>***

  - What else would you like to add to this question/ the questionnaire?
    Was möchten Sie noch zu dieser Frage/ dem Fragebogen sagen?

  - Are there annoying/ embarrassing/ inappropriate questions?
    Gibt es Fragen, die Sie verstörend/ ärgerlich/ peinlich/ unangemessen finden?

  - What questions or issues would you like to remove from the questionnaire?
    Welche Fragen oder Themen würden Sie gerne aus dem Fragebogen entfernen?

  - What questions or issues would you like to add?
    Welche Fragen oder Themen würden Sie gerne ergänzen?

  - How do you like the layout?
    Was sind Ihre Überlegungen zu der Aufmachung des Fragebogens?

-------------------THANK YOU! STOP RECORD/DANKE! UND AUFNAHME ANHALTEN --------------

---------------------------DEMO-FILE/ AUSFÜLLEN DES DEMOGRAPHIEBOGENS ----------------

---------------------------------DEBRIEFING ---------------------------------

- How are you? What can I do for you?
  Wie geht es Ihnen im Moment? – Was kann ich für Sie tun?
- Can we leave it like this right now?/ *Können wir es im Moment so lassen?*

FAREWELL. NOTIFICATION OF CONTACT AND AVAILABILIT
Verabschiedung. Hinweis auf Kontaktdaten.

Based on/ *Basierend auf*: Schildmann EK, Groeneveld EI, Denzel J, Brown A, Bernhardt F, Bailey K, et al. Discovering the hidden benefits of cognitive interviewing in two languages: The first phase of a validation study of the Integrated Palliative care Outcome Scale. Palliative medicine. 2015 Sep 28. PubMed PMID: 26415736.
